# Relationship between Tumor DNA Methylation Status and Patient Characteristics in African-American and European-American Women with Breast Cancer

**DOI:** 10.1371/journal.pone.0037928

**Published:** 2012-05-31

**Authors:** Songping Wang, Tiffany H. Dorsey, Atsushi Terunuma, Rick A. Kittles, Stefan Ambs, Bernard Kwabi-Addo

**Affiliations:** 1 Department of Biochemistry and Molecular Biology, Howard University, Washington, D.C., United States of America; 2 Laboratory of Human Carcinogenesis, Center of Cancer Research and Epidemiology and Genetics Research Program, National Cancer Institute, National Institutes of Health, Bethesda, Maryland, United States of America; 3 Department of Medicine, University of Illinois at Chicago, Chicago, Illinois, United States of America; Geisel School of Medicine at Dartmouth, United States of America

## Abstract

Aberrant DNA methylation is critical for development and progression of breast cancer. We investigated the association of CpG island methylation in candidate genes and clinicopathological features in 65 African-American (AA) and European-American (EA) breast cancer patients. Quantitative methylation analysis was carried out on bisulfite modified genomic DNA and sequencing (pyrosequencing) for promoter CpG islands of *p*16, *ESR*1, *RASSF*1A, *RARβ*2, *CDH*13, *HIN*1, *SFRP*1 genes and the *LINE*1 repetitive element using matched paired non-cancerous and breast tumor specimen (32 AA and 33 EA women). Five of the genes, all known tumor suppressor genes (*RASSF*1A, *RARβ*2, *CDH*13, *HIN*1 and *SFRP*1), were found to be frequently hypermethylated in breast tumor tissues but not in the adjacent non-cancerous tissues. Significant differences in the *CDH*13 methylation status were observed by comparing DNA methylation between AA and EA patients, with more obvious *CDH*13 methylation differences between the two patient groups in the ER- disease and among young patients (age<50). In addition, we observed associations between *CDH*13, *SFRP*1, and *RASSF*1A methylation and breast cancer subtypes and between *SFRP*1 methylation and patient's age. Furthermore, tumors that received neoadjuvant therapy tended to have reduced *RASSF*1A methylation when compared with chemotherapy naïve tumors. Finally, Kaplan Meier survival analysis showed a significant association between methylation at 3 loci (*RASSF*1A, *RAR*β2 and *CDH*13) and reduced overall disease survival. In conclusion, the DNA methylation status of breast tumors was found to be significantly associated with clinicopathological features and race/ethnicity of the patients.

## Introduction

Breast cancer is the most commonly diagnosed cancer among women in the United States, with >130,000 cases diagnosed yearly [1]. Among the different population groups within the United States, the overall breast cancer incidence is highest in European American (EA) women followed by African American (AA) women [2]. When breast cancer risk is stratified by age, EA women have the highest age-adjusted breast cancer incidence among women >50 years whereas in the <50 age group, the pattern is different with the EA women having lower incidence rates than AA [3]. In stark contrast to the incidence rates, AA women have generally higher age-adjusted mortality rates than EA women, accounting for the highest breast cancer mortality rates among all US population groups [3]. It has been suggested that the higher morality and lower survival rates among AA women are all due to factors associated with lower socioeconomic status and delayed disease diagnosis [4]–[7]. While socioeconomic factors are undoubtedly very important, they may not explain the full magnitude of the US survival health disparity in breast cancer [8]. Therefore, solving the racial disparity will require understanding possible tumor biological differences between population groups beyond the current knowledge of differences in hormone receptor status and disease grade. Breast cancer is a heterogeneous disease consisting of five major breast tumor subtypes: basal, human epidermal growth factor receptor 2 (HER2)-positive/estrogen receptor (ER)-negative, luminal A, luminal B, and normal like [9], [10]. Young AA women are at an increased risk to develop basal-like breast tumors, which tend to be both more aggressive and therapy resistant than the luminal type tumors and also have an increased propensity to metastasize to brain and lung [10]–[12]. AA women also experience almost twice the prevalence of the triple-negative disease (ER, progesterone receptor [PR], and human epidermal growth receptor 2 [HER2] negative) when compared with EA women [11], [13]. It has been hypothesized that the higher likelihood of developing triple-negative breast cancer might contribute to the higher mortality from breast cancer experienced by AA women compared with EA women [1], [13], [14]. Recent large scale gene expression profiling studies has confirmed the identities of these 5 subtypes based on their distinct gene expression signatures and clinical outcomes [10], [11], [15]. Genomic alterations that impact the expression patterns of individual genes or entire signatures in breast cancer have been described and include; inherited BRCA1 and BRCA2 [16], p53 [17], and H-ras-1 mutations [18] and overexpression of cyclin D1 [19]. Some of these genetic alterations tend to occur more commonly in tumors of AA than EA patients as shown for p53 and BRCA1/2 mutations [20], [21]. Moreover, Loo and coworkers observed significant differences in genomic copy number alterations in triple negative tumors from AA and EA women [22], whereas other investigators have reported differences in the gene expression profiles between AA and EA tumors in pathways related to tumor angiogenesis and chemotaxis [23].

In addition to somatic mutations, tumor DNA methylation pattern were found to show differences between breast cancer subtypes [24]–[27]. Another report also observed that hormone receptor-negative tumors from young AA patients show distinct DNA hypermethylation at certain loci when compared with EA tumors, suggesting candidate tumor biological differences between the two patient groups as they relate to cancer epigenetics [28]. The purpose of the present study was to test the hypothesis that tumor DNA methylation is different between AA and EA patients, as reported previously in one publication, and to characterize the relationship between the DNA methylation status of several breast cancer-related genes and patient characteristics or tumor markers.

## Materials and Methods

### Tissue

Fresh-frozen tissue samples corresponding to paired normal and breast tumor specimen from 65 patients (32 AA, and 33 EA women) were obtained from University of Maryland Medical Center (UMD) and Veterans Administration (VA) Hospital, Baltimore MD., and processed as previously described [29]. Clinical and pathological information (e.g., tumor receptor status) was obtained from medical records and pathology reports. Disease staging was performed according to the TNM system of the American Joint Committee on Cancer/Union Internationale Contre le Cancer (AJCC/UICC). The Nottingham system was used to determine the tumor grade. The collection of tumor specimens and clinical and pathological information was reviewed and approved by the University of Maryland Institutional Review Board for the participating institutions (UMD protocol 0298229). IRB approval of this protocol was obtained at all institutions (UMD, VA and Howard University). The tissues were taken as distant as possible during routine surgery and none of the adjacent normal tissues contained visible tumor contamination by histological analysis. High molecular weight genomic DNA was isolated from fresh-frozen breast tissues using the DNeasy® isolation kit following the manufactures directions for purification from human tissues (Qiagen, Valencia, CA). DNA concentration was measured using the Nanodrop Spectrophotometer (Nanodrop, Wilmington, DE).

### Bisulfite modification, PCR, and pyrosequencing analysis

High molecular weight genomic DNA extracted from breast tissues was modified using sodium bisulfite treatment [30]. Briefly, genomic DNA (2 ìg) was denatured in 0.3 mol/L NaOH at 37°C for 15 minutes; sodium bisulfite and hydroquinone were added to final concentrations of 3.1 mol/L and 0.5 mmol/L, respectively. The reaction was incubated at 50°C for 16 hours and desalted using Wizard DNA purification resin (Promega) according to the instruction of the manufacturer. Bisulfite modification was completed by DNA desulfonation in 0.3 mol/L NaOH at 37°C for 15 minutes. Modified DNA was precipitated with ethanol, washed in 70% ethanol, dried, and dissolved in 50 ìL of TE buffer. The PCR primers were designed to assay the methylation status of CpGs within 0.5 kb from the transcription start site. The CpG islands interrogated were previously described; *p*16, *RASSF*1A, *RARβ*2 and *ESR*1 [31], [32], *LINE*1, *CDH*13, *HIN*1 [33] and *SFRP*1 [34]. PCR primer sequences and sequencing primer sequences are listed in the Table S1. Either one-step or two-step PCR reactions were carried out using 2 ìL of bisulfite-converted genomic DNA and either one or two sets of different bisulfite PCR primers in a standard PCR reaction mix. One of the primers (reverse primer) in the final PCR reaction was biotinylated to create an ssDNA template for the pyrosequencing reaction. Where indicated, we used a previously described amplification protocol [35] based on the universal primer approach. Briefly, the biotinylated reverse primer was substituted with a 5′ tailed unlabeled reverse primer and a biotinylated universal primer at a ratio of 1∶9 in the PCR reaction. The integrity of the PCR product was verified on 1.5% agarose gels with ethidium bromide staining. The PCR product was immobilized on streptavidin-Sepharose beads (Amersham), washed, and denatured, and the biotinylated strands were released into annealing buffer containing the sequencing primer. Pyrosequencing was done using the PSQ HS96 Gold SNP Reagents on a PSQ 96HS machine (Qiagen). Bisulfite-converted DNA from blood of normal volunteers and blank reactions, with water substituted for DNA, served as negative control and bisulfite-converted *Sss*I methylase–treated blood DNA served as a positive control. Each bisulfite PCR and pyrosequencing reaction was done at least twice. A typical example of methylation raw data presented as pyrogram for CDH13 CpG island is shown in Pyrogram Data S1.

### Statistical analysis

Statistical analyses were performed using R, a language and environment for statistical computing by R Development Core Team at R Foundation for Statistical Computing, and packages in Bioconductor [36]. Specifically, survival analysis was performed using survival package. Z scores for DNA methylation levels of each gene was calculated across all tumor samples. Groups of samples were compared by Mann-Whitney test with Bonferroni correction.

## Results

### Methylation status of gene promoter CpG islands in matched normal and breast cancer tissues

**Figure 1:**
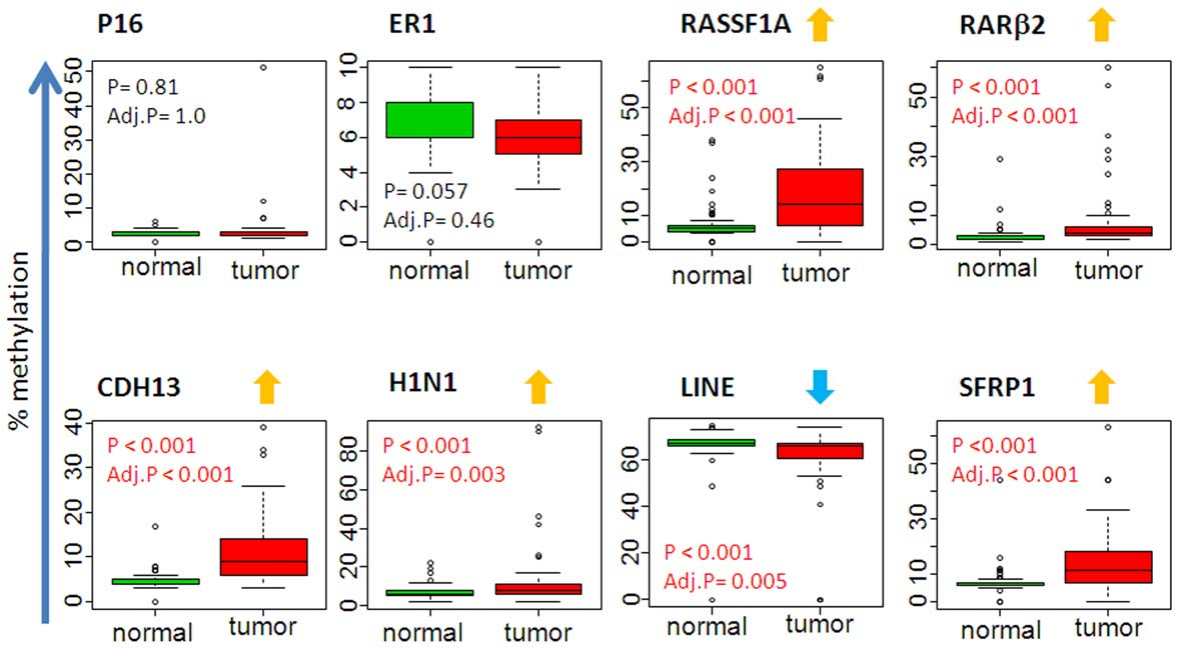
Quantitative DNA methylation analysis in human breast tissues. The percentage of DNA methylation levels at promoter CpG islands were analyzed in bisulfite-modified genomic DNA extracted from matched pairs of non-cancerous (normal) and breast tumors (tumor) tissue samples obtained from AA and AE cancer patients. *Y* axis, percentage of methylated cytosines in the samples as obtained from pyrosequencing. *X* axis, normal and tumor tissues obtained from AA and EA. *P* value is indicated for each gene (Mann-Whitney). Adj. P: adjusted P value (Bonferroni).

We evaluated the DNA methylation status for a panel of genes in paired adjacent non-cancerous and breast tumor specimens from a total of 65 patients (32 AA and 33 EA women). The demographic, clinicopathological characteristics and percent ancestry of the patient samples are presented in Table 1. The clinicopathological features did not differ significantly between the two patient groups by age at diagnosis, ER status, tumor size and disease stage, lymph node status or tumor p53 status. There are equal numbers of AA and EA patients with triple-negative or basal-like tumors in the data set. AA patients tended to have higher grade tumors more frequently than EA patients. We investigated the methylation status of 8 genes of which 6 are known growth suppressor genes namely *p*16, *RASSF*1A, *RARβ*2, *CDH*13, *HIN*1 and *SFRP*1 genes, with the other 2 encoding the estrogen receptor alpha (*ESR*1) or representing a global methylation marker, the *LINE*1 repetitive elements [31]–[34]. We used pyrosequencing assays to analyze the methylation status of these genes in the 65 matched tissue pairs. For each gene studied, the percentage (%) of methylation at a specific promoter was compared between the non-cancerous and the cancerous tissues (Fig. 1). The pyrosequencing analysis was repeated only with cases that were not exposed to neoadjuvant therapy (Fig S1). There was little difference from all cases analyzed. Five of the 8 genes (*RASSF*1A, *RARβ*2, *CDH*13, *HIN*1 and *SFRP*1) were frequently hypermethylated in the tumor samples. *ESR*1 was uncommonly methylated and the *p*16 gene was not methylated in either tumor or non-cancerous breast tissues. The *LINE*1 repetitive element, a global methylation marker, showed a distinct methylation pattern and was found to be hypermethylated in both the malignant and non-cancerous breast tissue samples, as one may expect with a significant reduction of % *LINE*1 methylation in tumors compared to non-cancerous tissue samples. To further examine the relationship between the methylation profile with patient characteristics and tumor markers, we focused on the 4 genes (*RASSF*1A, *RARβ*2, *CDH*13 and *SFRP*1) that showed the highest relative changes in tissue methylation status across the 65 tissue pairs.

**Table 1:** Demographic, clinicopathologic characteristics and percent ancestry of breast cancer patient cases.

		AllN = 67	AA^1^N = 32	EA^2^N = 35	*P*-value^3^
		Mean ± SD			t test
Age (years)		54±15.8	52.9±16.1	54.9±15.7	0.62
Tumor size (cm across) (n = 60)		4.2±2.7	3.8±2.3	4.5±3.0	0.25
West-African ancestry (%) among AA (range: 67% to 95%; n = 32)			83.2±8.7	(16.0±8.8)	<0.001
European ancestry (%) among EA (range: 75% to 100%; n = 35)			(2.2±0.5)	97.6±4.7	<0.001
		N	N	N	Fisher's exact test
**ER Status**	**Negative**	34	16	18	
	** Triple-negative and/or basal-like** 4	16	8	8	
	** HER2-positive**	11	8	3	
	**Positive**	33	16	17	1.0^5^
	** Luminal A**	21	12	9	
	** Luminal B (ER/HER2-positive)**	10	3	7	
**TNM Stage**	**I**	6	2	4	
	**II**	46	24	22	
	**III**	15	6	9	0.63
**Grade**	**1**	8	1	7	
	**2**	20	8	12	
	**3**	29	18	11	0.029^6^
	**Unknown**	10	5	5	
**p53 mutation**	**Negative**	51	25	26	
	**Positive**	16	7	9	0.78
**Menopause**	**No**	26	14	12	
	**Yes**	33	14	19	0.44^6^
	**Unknown**	8	4	4	
**Income**	**Less than $15,000**	12	10	2	
	**$15,000 to $60,000**	26	13	13	
	**More than $60,000**	11	1	10	<0.01^6^
	**Unknown**	18	8	10	
**Body mass index**	**≤24.9**	18	7	11	
	**25.0 to 29.9**	15	5	10	
	**≥30.0**	29	17	12	0.25^6^
	**Unknown**	5	3	2	
**Neoadjuvant therapy**	**No**	53	30	23	
	**Yes**	9	1	8	0.026^6^
	**Unknown**	5	1	4	

SD = standard deviation.

1 AA = African-American,

2 EA = European-American. Race/ethnicity is determined by self-identification;

3 AA versus EA;

4 basal-like = ER-negative, HER2-negative, and either cytokeratin 5/6-positive or EGFR-positive;

5 ER-negative versus ER-positive;

6 Unknown not included.

### Correlation between promoter methylation and gene expression level

**Figure 2:**
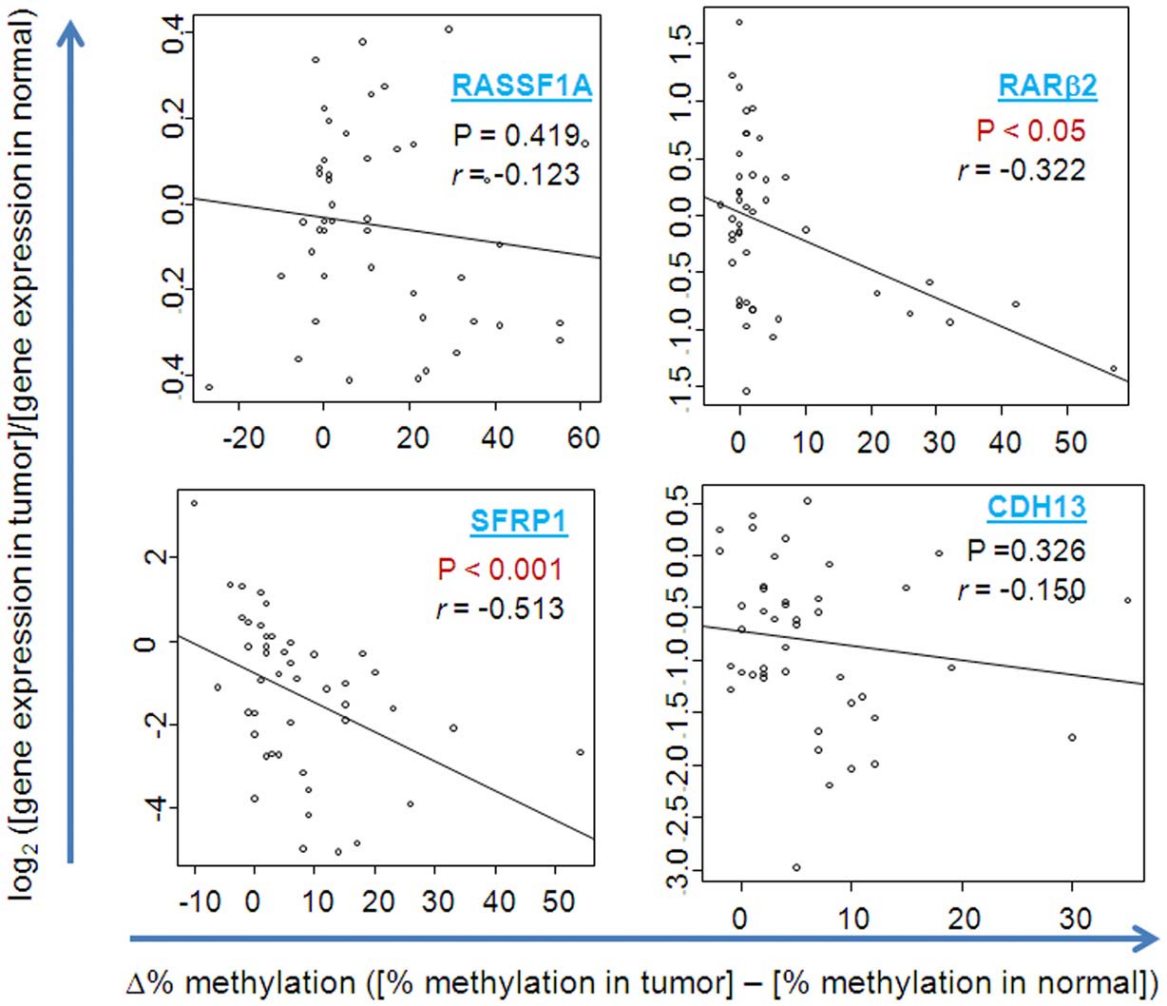
Promoter methylation and gene expression. The ratio of gene expression data of tumor to non-cancerous (normal) from a genome-wide gene expression microarray [23] was correlated with the methylation difference between the tissue pairs. The log 2 ratio of gene expression in matched pair tumor and normal is shown on Y axis. The relative methylation frequency is shown on the X-axis. *P* value and the correlation index; *r* (Rho) values are from Spearman rank correlation analysis.

To assess the biological significance of promoter methylation in the breast tumor samples, we correlated the relative methylation frequency with gene expression data from a previously described genome-wide gene expression study [23]. As shown in figure 2, the Spearman rank correlation revealed significant inverse association between promoter methylation and gene expression for RARβ2 (*r* = −0.322; p<0.05) and SFRP1 (*r* = −0.513; p<0.001), suggesting that silencing of these genes is at least partly caused by promoter methylation. We did not observe a significant association between methylation and gene expression for the RASSF1A and CDH13 loci, suggesting that other repression mechanisms like chromatin silencing or methylation at other CpG island are important in RASSF1A and CDH13 silencing.

### Relationship between gene methylation status and patient characteristics

**Figure 3:**
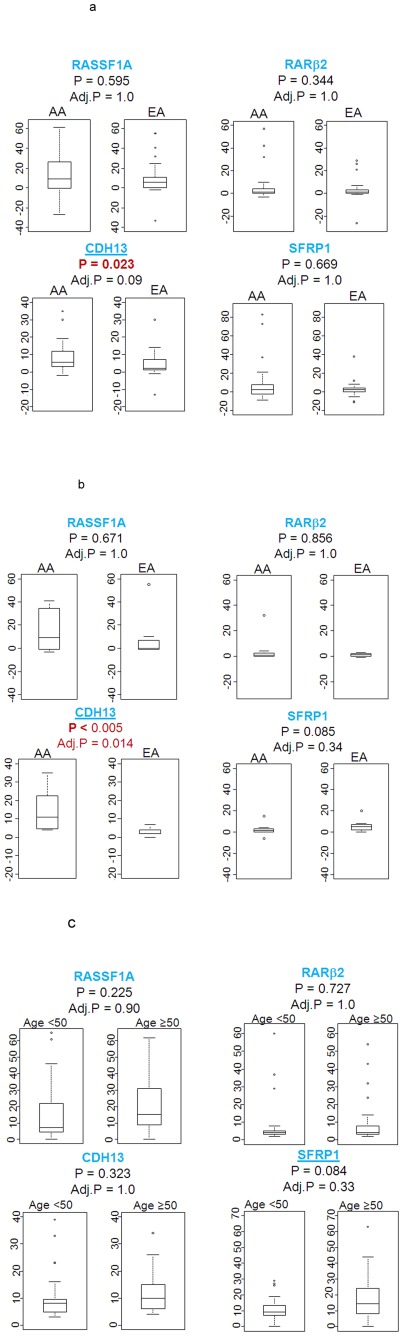
Gene methylation status and patient characteristics. **A.** Relative methylation frequency (% methylation in tumor minus % methylation in adjacent normal) stratified by race; AA (n = 32) and EA (n = 33; Mann-Whitney). Adj. P: adjusted P value (Bonferroni) **B.** Relative methylation frequency stratified by ER- negative (−) patients with age<50 (17 cases in total; 8 AA cases and 9 EA cases) P value is shown (Mann-Whitney). **C.** Percentage methylation frequency in breast tumor cases stratified by age (age<50: n = 31; age≥50: n = 34). P value is shown (Mann-Whitney).

To further explore the association of the tumor methylation status with patient characteristics such as age, race/ethnicity and ER status, we initially examined gene methylation differences between AA and EA women (Fig. 3a). The statistical analysis revealed difference in the methylation status between AA and EA patients for the *CDH*13 gene (p = 0.023), but not for any other gene. We repeated the analysis for AA and EA women that were not exposed to neoadjuvant therapy, there was little difference from all cases analyzed (Fig S2a). When we studied the relative methylation frequency after stratification into two age groups with the cutoff point at age 50, we found that young (<50 years of age) and ER-negative AA patients had a significantly higher methylation index at the *CDH*13 locus than the matched EA patients (p<0.005; Fig. 3b), while the analysis of *RASSF*1A, *RARβ*2 and *SFRP*1 did not find significant differences in this subgroup analysis. In contrast, we could not detect any methylation differences between AA and EA patients in the ER-positive disease, indicating that the CDH13 differences between the two patient groups are restricted to ER-negative tumors. These findings are consistent with findings by others that methylation differences in breast tumors between AA and EA patients are perhaps restricted to ER-negative tumors [28]. To ascertain whether individual ancestry is influencing the methylation status, we carried out a correlation analysis between West African score (for AA) or European score (for EA) and methylation score (Fig S2b). There was no significant correlation between CDH13 methylation and individual ancestry score suggesting that no functional African ancestry related Single nucleotide polymorphic marker(s) is affecting CDH13 methylation. Lastly, age itself was modestly associated with the methylation status at the *SFRP*1 locus in the tumor samples tumor samples (p = 0.084; Fig. 3c). This change is not correlated with normal samples was there was little variation in % methylation in normal tissues (as shown in Fig. 1). Overall, we observed the trend that tumors of older patients tend to have increased promoter methylation.

### Relationship between gene methylation and tumor subtypes

**Figure 4:**
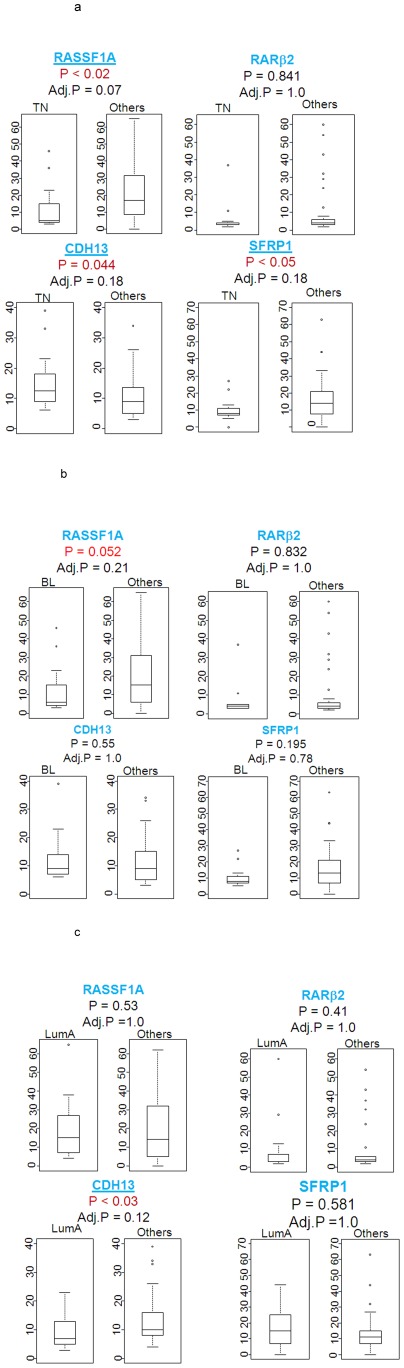
Gene methylation status and tumor subtypes. Percentage methylation frequency stratified by subtype: **A.** Triple-negative (TN) tumors (n = 14) versus others (n = 43), **B.** Basal-like (BL) tumors (n = 13) versus others (n = 45). **C.** Luminal A (LumA) tumors (n = 21) versus others (n = 37; Mann-Whitney).

Next, we analyzed tumor characteristics to investigate whether there is a correlation of % methylation with clinical outcomes (Fig. 4a–c). We observed that triple-negative breast cancers tended to have increased *CDH*13 methylation (p = 0.044) and a decreased methylation of the *RASSF*1A (p<0.02) and SFRP1 (p<0.05, Fig. 4a) genes when compared with other subtypes. The closely related basal-like tumors also had decreased *RASSF*1A methylation (p = 0.052; Fig. 4b) when compared with other breast cancer subtypes, while the luminal A tumors tended to have a reduced *CDH*13 methylation (p<0.03; Fig. 4c).

### Correlating gene methylation changes with neoadjuvant therapy

**Figure 5:**
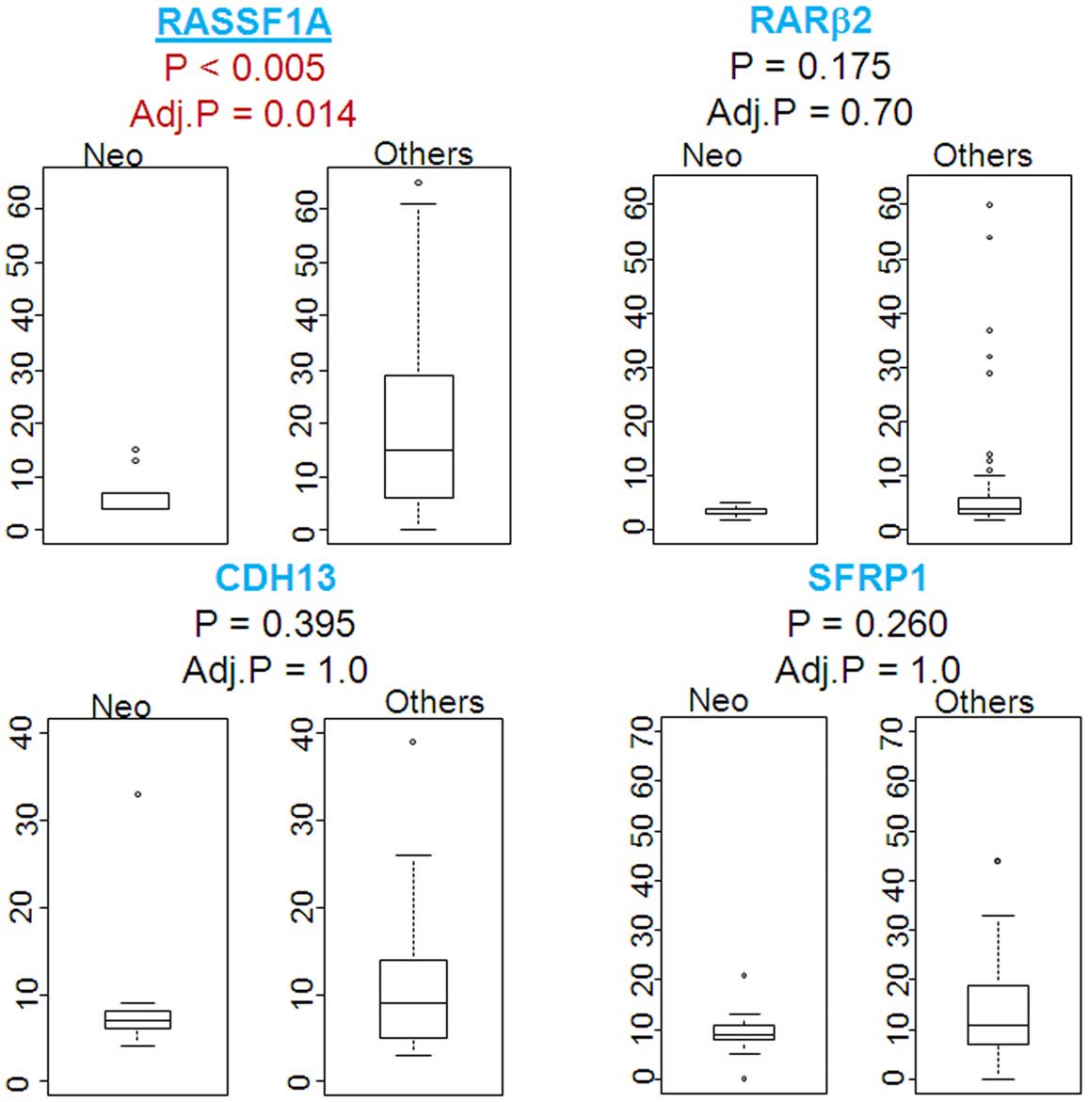
Percentage methylation frequency and neoadjuvant therapy. The % methylation in tumors with neoadjuvant therapy (n = 9−2 luminal; 4 basal-like and 3 triple-negative cases) and without (n = 53; Mann-Whitney). There were 3 cases for which neoadjuvant therapy data was not available.

We investigated the association of relative methylation changes in breast tumors with neoadjuvant therapy by comparing tumors that received neoadjuvant therapy (n = 9) to those that did not (n = 53; Mann-Whitney). Tumors that received neoadjuvant therapy tended to have reduced *RASSF*1A methylation when compared with tumors that did not (p<0.005; Fig. 5), suggesting that neoadjuvant therapy may affect *RASSF*1A methylation.

### Methylation signatures that are associated with survival and ethnic difference

**Figure 6:**
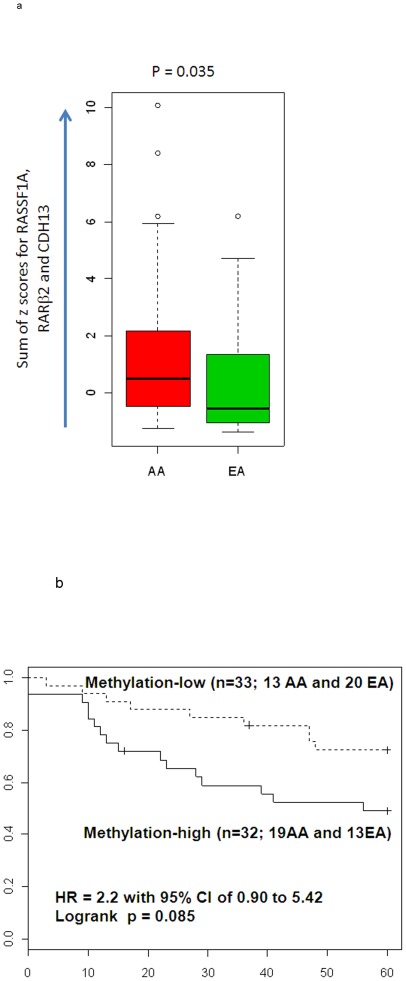
Methylation signatures and survival. **A.** The sum of z-scores for RASSF1A, RARβ2 and CDH13 were significantly higher in AA patients (p = 0.035; Mann-Whitney; n = 65). **B.** Patients with high values for the sum of Z scores demonstrated poor outcome (p = 0.085; log rank test; n = 65, adjusted for age at diagnosis, and ER status). Hazard ratio was 2.20 with the 95% confidence interval of 0.90 to 5.42, adjusted for age at diagnosis and ER status. The median of the sum of z-scores for all cases was used as the cutoff.

We investigated the association between DNA methylation status and outcome of the patients. While the DNA methylation status of individual genes were not associated with patients survival (data not shown), we tested if the combination of genes could improve the ability in detecting the association between methylation status and disease outcome. We integrated the DNA methylation status of multiple genes by summing their methylation levels as z-scores. When z-scores of three genes that tended to be methylated more in AA patients (*RASSF*1A, *RARβ*2 and *CDH*13; shown in Fig. 3B) were combined, the sum of methylation scores become significantly higher in AA tumors (p = 0.035; Fig. 6A). Furthermore, patients with high methylation scores revealed poor outcome (Fig. 6B), implying the potential association between methylation of these genes and poor outcome of AA patients.

## Discussion

Many existing data indicates that aberrant DNA methylation is associated with breast cancer risk; however, to our knowledge there is only one published report to suggest an association between DNA methylation changes and invasive ductal breast cancers comparing AA patients with EA patients [28]. In the present study, we used quantitative DNA methylation analysis to measure the methylation frequency in 6 known tumor suppressor genes, the estrogen receptor alpha and 1 global methylation marker to delineate their methylation differences in AA and EA patients and to characterize the relationship between the DNA methylation status and patient characteristics or tumor markers. Our most notable observation is the presence of a significantly higher methylation at a CpG site in the *CDH*13 gene in breast tumor samples from AA women when compared with EA women among young and ER-negative patients. The *CDH*13 gene is a well-known tumor suppressor gene whose protein product is a putative mediator of cell-cell interaction and cancer cell invasion and metastasis [37]. Thus *CDH*13 hypermethylation may contribute to the distinct molecular alterations hypothesized for AA and EA tumors that may play a role in the early onset of breast cancer that lacks ER expression. In humans, methylation changes are more and more recognized as part of pathologic aging physiology [31], [38]. While associations between gene promoter methylation and age have been reported, there is inconsistency in findings from different groups. We observed that age was significantly associated with methylation of *SFRP*1 gene and may affect AA differently than EA women. Our data offer the possibility that gene methylation pattern in breast tissues may contribute to altered gene expression in older AA and EA individuals.

Recent microarray profiling of invasive breast carcinomas has revealed 5 distinct tumor subtypes with distinct gene expression signatures and clinical outcomes. Because AA and EA women with breast cancer experience differences in tumor subtype presentations, we investigated methylation changes as potential biomarkers for tumor subtypes. In the 65 cases that we tested, 14 cases were triple-negative tumors and tended to have increased *CDH*13 methylation and a decreased methylation of the *RASSF*1A and *SFRP*1 genes. The basal-like tumors (13 cases) tended to have a decrease RASSF1A methylation whereas the luminal A tumors (21 cases) tended to have a decrease CDH13 methylation. Our data suggests that DNA methylation reflected by the *CDH*13, *RASSF*1A and *SFRP*1 locus panel might have a role in the phenotype of breast tumor subtypes. We did not see any correlation of methylation with lymph node, tumor grade which may mainly reflect a limitation on the sample size used in this study.

Methylation of specific candidate genes or groups of genes has been associated with poorer prognosis and these genes may affect tumor aggressiveness independent of their methylation phenotype [39]–[41]. Such candidate genes are being explored as potential biomarkers for detecting breast cancer invasion and for predicting response to neoadjuvant therapy as well as other therapeutic intermediates. We observed that neoadjuvant therapy may affect *RASSF*1A methylation suggesting that *RASSF*1A methylation could actually be a useful predictive marker for neoadjuvant therapy response. Finally, high methylation of *RASSF*1A, *RARβ*2 and *CDH*13 loci were associated with worse overall disease survival in our analysis. These 3 loci tended to be more methylated in AA compared with EA tumors supporting our hypothesis that differences in methylation patterns may contribute to the more aggressive and poorer disease outcome in AA women.

Consistent differences in the methylation pattern between AA and EA breast cancer cases can be attributed to environmental factors including dietary factors (e.g., availability of methyl donors), or functional SNP allele frequencies. Diet-low levels of S-adenosyl methionine (SAM), and the production of SAM depends on dietary factors such as folate, vitamin B6, and vitamin B12. The main role of folate is to provide one-carbon units in several reactions necessary for DNA methylation and synthesis, while vitamins B12 and B6 serve as cofactors in some of these reactions [42]. Sustained low levels of these nutrients may lead to disturbance in DNA methylation, synthesis, and repair, possibility influencing breast cancer disparity.

In conclusion, ongoing studies clearly demonstrates differential DNA methylation patterns in AA and EA breast cancer cases that may represent an integration of lifestyle and genetic predisposing factors resulting in altered patterns of gene expression and differences in clinical outcome and behavior.

## Supporting Information

Figure S1 **Quantitative DNA methylation analysis in human breast tissues not exposed to neoadjuvant therapy.** The percentage of DNA methylation levels at promoter CpG islands were analyzed in bisulfite-modified genomic DNA extracted from matched pairs of non-cancerous (normal) and breast tumors (tumor) tissue samples obtained from AA and AE cancer patients. *Y* axis, percentage of methylated cytosines in the samples as obtained from pyrosequencing. *X* axis, normal and tumor tissues obtained from AA and EA. *P* value is indicated for each gene (Mann-Whitney). Adj. P: adjusted P value (Bonferroni).
(TIF)

Figure S2 **Correlating gene methylation status and patient characteristics.** **A.** Gene methylation status in patients not exposed to neoadjuvant therapy. Relative methylation frequency (% methylation in tumor minus % methylation in adjacent normal) stratified by race; AA (n = 32) and EA (n = 33; Mann-Whitney). Adj. P: adjusted P value (Bonferroni). **B.** Correlation of gene methylation and individual ancestry score. Relative CDH13 methylation frequency stratified by ER- negative (−) patients with age <50 (17 cases in total; 8 AA cases and 9 EA cases) was correlated with individual informative markers for West African and European ancestry. P value is shown (Mann-Whitney).
(TIF)

Pyrogram Data S1 **Representative pyrogram traces for CDH13 CpG island.** Grey columns represent regions of C to T polymorphic sites. Representative matched normal and prostate tumor samples for African-American (AA) and European-American (EA) breast cancer patients are shown. Top, percentage methylation at each CpG sites. Y-axis signal peaks proportional to the number of nucleotides incorporated. X-axis, the nucleotide incorporated in the pyrosequencing reaction.
(TIF)

Table S1 **Primer sequences used in the pyrosequencing analysis.** U represents universal primer sequence- GGGACACCGCTGATCGTTTA.
(DOC)
