# Supplementary figures and images for: Relationship between Tumor DNA Methylation Status and Patient Characteristics in African-American and European-American Women with Breast Cancer
Source: PLoS One. 2012 May 31;7(5):e37928. doi: 10.1371/journal.pone.0037928 (PMC3365111; doi:10.1371/journal.pone.0037928)

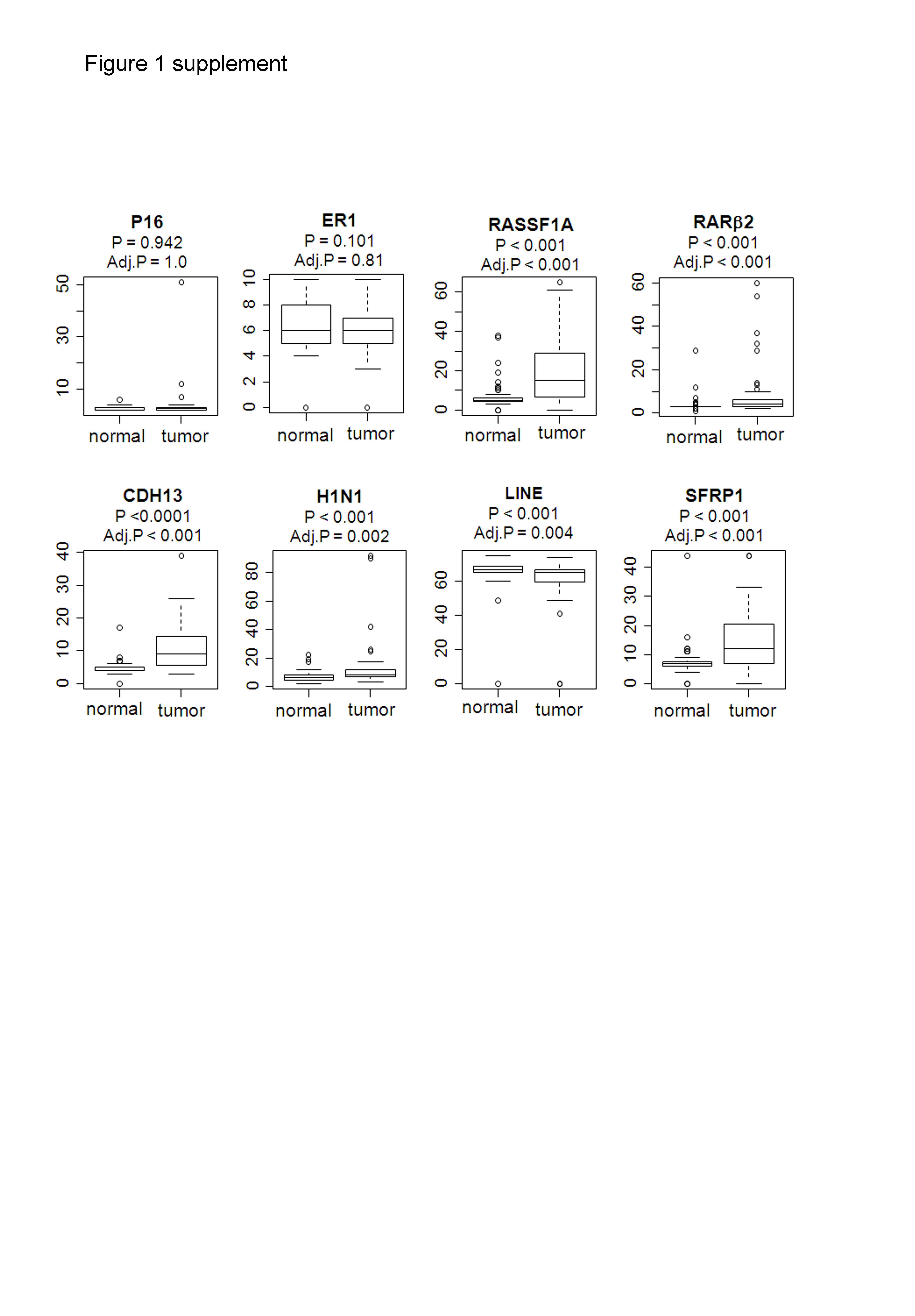

Supplement: Figure S1 — Quantitative DNA methylation analysis in human breast tissues not exposed to neoadjuvant therapy. The percentage of DNA methylation levels at promoter CpG islands were analyzed in bisulfite-modified genomic DNA extracted from matched pairs of non-cancerous (normal) and breast tumors (tumor) tissue samples obtained from AA and AE cancer patients. Y axis, percentage of methylated cytosines in the samples as obtained from pyrosequencing. X axis, normal and tumor tissues obtained from AA and EA. P value is indicated for each gene (Mann-Whitney). Adj. P: adjusted P value (Bonferroni). (TIF) [file pone.0037928.s001.tif]

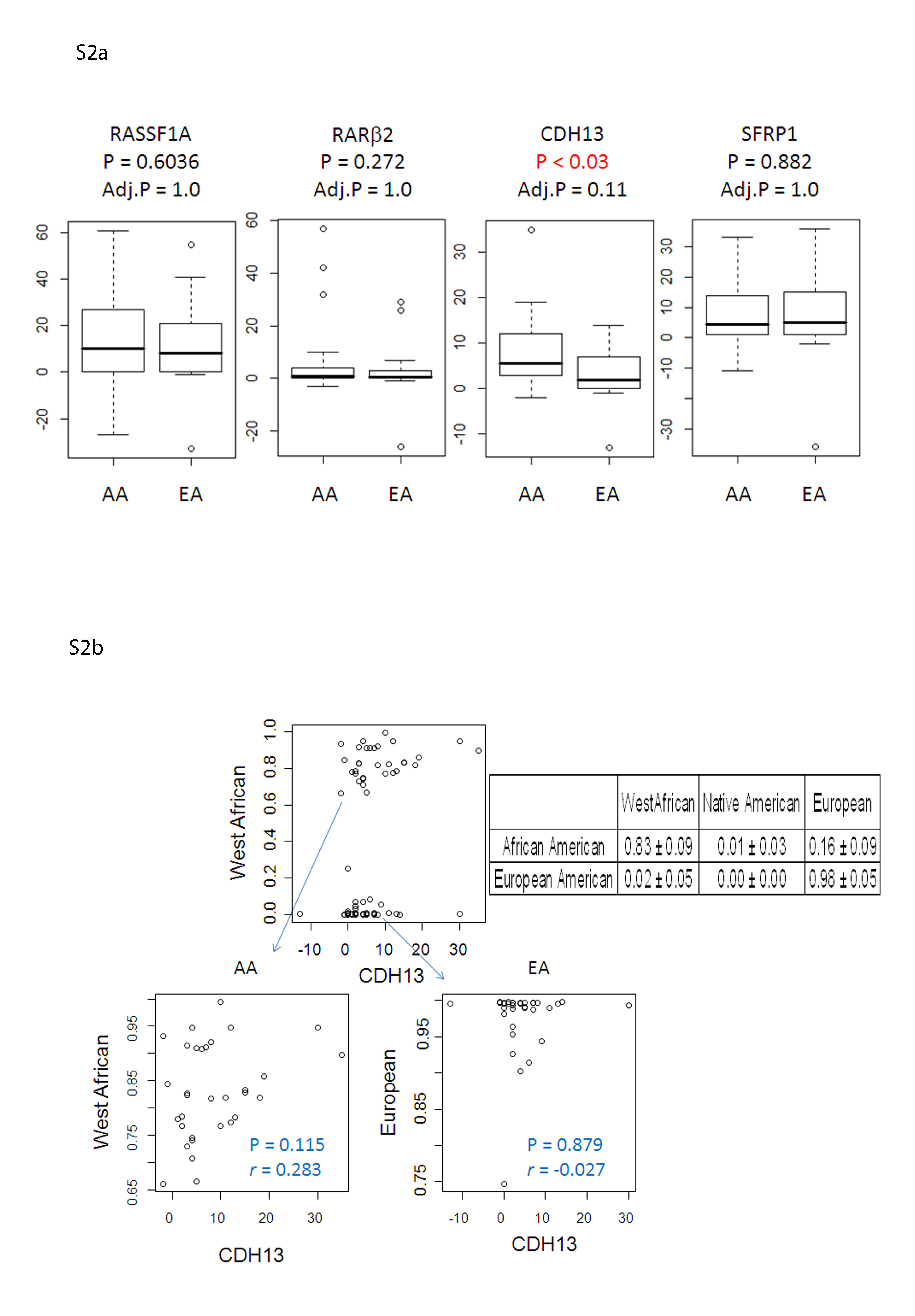

Supplement: Figure S2 — Correlating gene methylation status and patient characteristics. A. Gene methylation status in patients not exposed to neoadjuvant therapy. Relative methylation frequency (% methylation in tumor minus % methylation in adjacent normal) stratified by race; AA (n = 32) and EA (n = 33; Mann-Whitney). Adj. P: adjusted P value (Bonferroni). B. Correlation of gene methylation and individual ancestry score. Relative CDH13 methylation frequency stratified by ER- negative (−) patients with age <50 (17 cases in total; 8 AA cases and 9 EA cases) was correlated with individual informative markers for West African and European ancestry. P value is shown (Mann-Whitney). (TIF) [file pone.0037928.s002.tif]

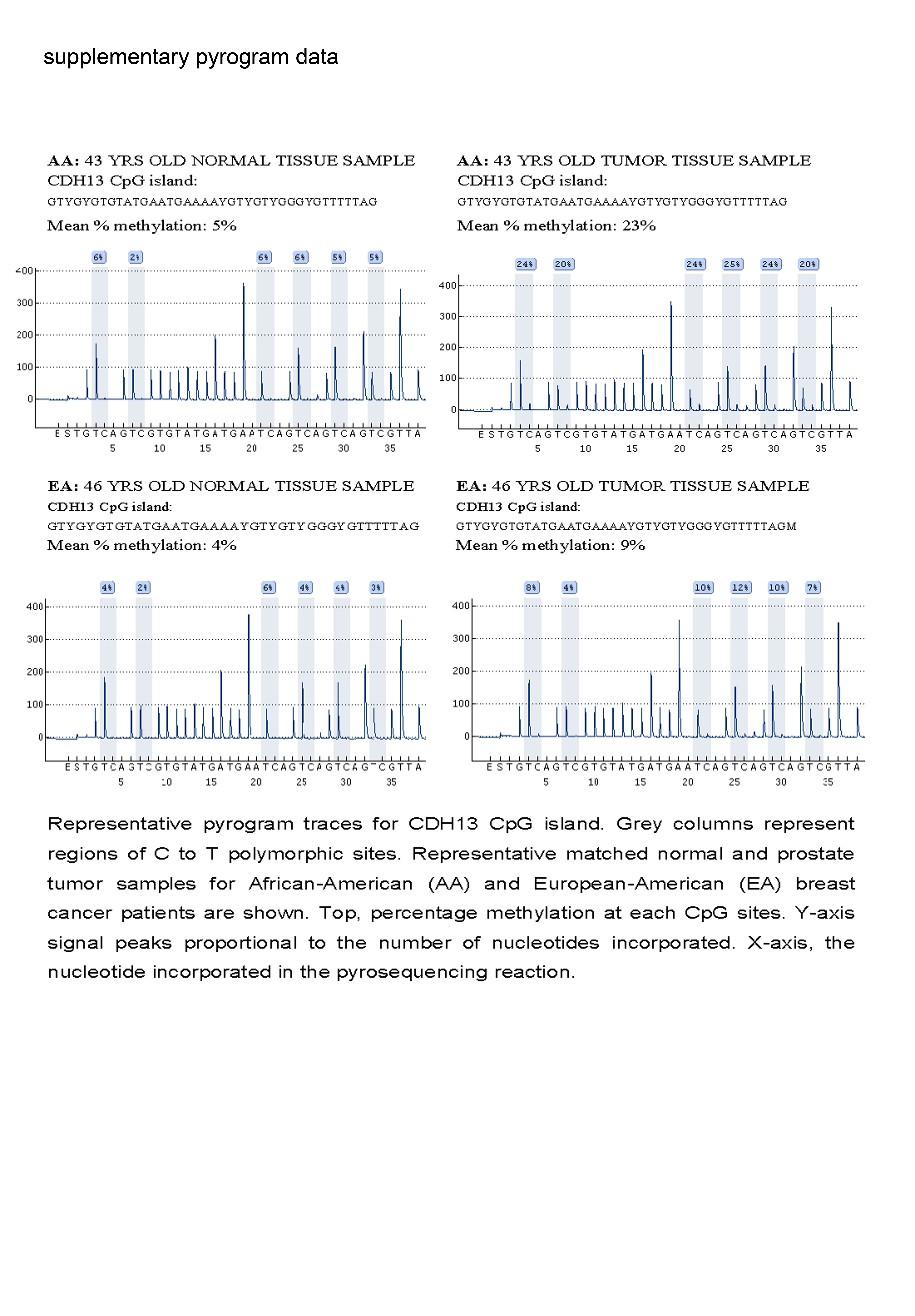

Supplement: Pyrogram Data S1 — Representative pyrogram traces for CDH13 CpG island. Grey columns represent regions of C to T polymorphic sites. Representative matched normal and prostate tumor samples for African-American (AA) and European-American (EA) breast cancer patients are shown. Top, percentage methylation at each CpG sites. Y-axis signal peaks proportional to the number of nucleotides incorporated. X-axis, the nucleotide incorporated in the pyrosequencing reaction. (TIF) [file pone.0037928.s003.tif]
