# Supplementary material for: Relationship between Tumor DNA Methylation Status and Patient Characteristics in African-American and European-American Women with Breast Cancer
Source: PLoS One. 2012 May 31;7(5):e37928. doi: 10.1371/journal.pone.0037928 (PMC3365111; doi:10.1371/journal.pone.0037928)
Supplement: Table S1 — Primer sequences used in the pyrosequencing analysis. U represents universal primer sequence- GGGACACCGCTGATCGTTTA. (DOC) [file pone.0037928.s004.doc]

| **Pyrosequencing** |  |  |  |
| --- | --- | --- | --- |
|  | **Sense** | **Antisense** | **Sequencing primer** |
| CDH13 (1^st^ Step) | TTTGGGAAGTTGGTTGGTTG | ACAACCCCTCTTCCCTACCT | AGGAAAATATGTTTAGTGTA |
| CDH13 (2^nd^ Step) | AGTTTGGTTTTTAAGGAAAATATGTTTAGT | Biotin-AACCAAATTCTCCACTACATTTTATCC |  |
| *LINE*1 | TTTTGAGTTAGGTGTGGGATATA | Biotin-AAAATCAAAAAATTCCCTTTC | AGTTAGGTGTGGGATATAGT |
| HIN1 (1^ST^ Step) | GGGGAGTTTATAGGAGTTGTAGGATAG | AACCAAACCAACAAAACTTTCTCAA | GGGTTAAGTAGAGTTTTAGGAG |
| HIN1 (2^ND^ Step) | GGGGAGTTTATAGGAGTTGTAGGATAG | U-ACCAAAACCCAATATAAAAAACCT  5’-Biotin-U |  |
| RARβ2 (1^st^ Step) | AGTTGGGTTATTTGAAGGTTA | TACCCAAACAAACCCTACTC and U-CCCAAACAAACCCTACTC | GGGACACCGCTGATCGTTTA |
| RARβ2 (2^nd^ Step) | AAGTAGTAGGAAGTGAGTTGTTTAGA | 5’-Biotin-U |  |
| | RASSF1A |  | | --- | --- | | GGGGGAGTTTGAGTTTATTGA | BIOTIN-CTACCCCTTAACTACCCCTTCC | GGGTAGTATTAGGTTGGAG |
| p16 | GGTTGTTTTCGGTTGGTGTTTT | BIOTIN-ACCCTATCCCTCAAATCCTCTAAAA | TTTTGTTTGGAAAGAT |
| SFRP1 (1^ST^ Step) | GGGGAATTTGTTATATTTAAGTATTT | ATACCCCTACTCAACAAAAACTACC | AGTAGAAGTAGAAGAATTGT |
| SFRP1 (2^nd^ Step) | GGGGAATTTGTTATATTTAAGT | U-ACACCCAAATCTTCCTCTA  and  5’-Biotin-U |  |
| ER1 | TGTGTTTTTTTTTTAGGTGG | BIOTIN- AACCATCCCAAATACTTTAATA | GGATACGGTTTGTATTTTG |

Table S1. Primer sequences used in the pyrosequencing analysis.

U represents universal primer sequence- GGGACACCGCTGATCGTTTA
